# Impact of Statewide Investment in Alzheimer's Disease and Related Dementias in Georgia

**DOI:** 10.1002/alz70860_107701

**Published:** 2025-12-23

**Authors:** Antoine R Trammell, Rebecca McIntosh, Alexis A Bender, Miranda A Moore, James J. Lah, Chadwick M Hales

**Affiliations:** ^1^ Emory University Goizueta Alzheimer's Disease Research Center, Atlanta, GA, USA; ^2^ Emory University, Atlanta, GA, USA; ^3^ Goizueta Alzheimer's Disease Research Center, Emory University, Atlanta, GA, USA; ^4^ Emory University School of Medicine, Atlanta, GA, USA

## Abstract

**Background:**

By 2050, 14 million Americans are projected to have Alzheimer's disease and related dementias (ADRD). Among older adults, African American (AA) have a higher risk than their non‐Hispanic White (NHW) counterparts. Despite higher ADRD risk, AA adults are less often evaluated in specialized memory disorder clinics. Besides the National Plan to Address Alzheimer's Disease, Building our Largest Dementia (BOLD) is a federal initiative to address ADRD prevention and early detection through public health programs at the local and state levels. BOLD eligibility funding requires developing a state plan to address ADRD. ADRD advocates across academic institutions, community organizations, and state leadership collaborated to create the Georgia Alzheimer's and Related Dementias State Plan (GARD) in 2014. As part of the state plan, Georgia's legislature funded the Georgia Memory Net (GMN) in 2018 to create five specialized memory assessment clinics for screening, diagnostic care, and caregiver resource connections for all Georgians with cognitive impairments, making Georgia one of several states with an established program to address ADRD‐related challenges. This project explores GMN's impact since its inception by comparing aggregated data with Centers for Medicare and Medicaid Services (CMS) fee‐for‐service data.

**Method:**

We summarized aggregated demographic, location, and diagnosis data from GMN clinics from 2017 to 2024. Then, using chi‐square, we compared data from GMN clinics with statewide CMS FFS data.

**Result:**

From 2017 to 2024, GMN clinics evaluated 2,426 patients. By location, GMN clinic providers evaluated more Georgians living in urban (81%) vs. rural (19%) areas. Among diagnoses, most people seen at GMN clinic had mild cognitive impairment (55%). Per CMS FFS data, 3,764,021 Georgians underwent cognitive evaluations. By demographics, compared to statewide CMS FFS claims, GMN clinic sites evaluated more older AA adults (57% vs. 29%, *p* <0.01).

**Conclusion:**

Increasing access to specialized memory disorder clinics, including among older AA adults, a population with higher ADRD risk. Further, more people seen at GMN clinics were diagnosed at earlier disease stages. These results show the potential for statewide ADRD investments to impact ADRD disparities and disease diagnosis at earlier stages.

